# Availability and access to long-term inpatient rehab centers in north Tshwane, Gauteng

**DOI:** 10.4102/hsag.v30i0.3012

**Published:** 2025-11-21

**Authors:** Rorisang G. Komane-Mnguni, Nonhlanhla M. Mkhize, Tshepiso Mfolo, Thomas K. Madiba

**Affiliations:** 1 School of Health Systems and Public Health, Faculty of Health Sciences, University of Pretoria, Pretoria, South Africa; 2 Department of Orthodontics, School of Dentistry, Faculty of Health Sciences, University of Pretoria, Pretoria, South Africa; 3 Department of Community Dentistry, School of Dentistry, Faculty of Health Sciences, University of Pretoria, Pretoria, South Africa

**Keywords:** treatment services, substance abuse treatment, access, Tshwane, rehabilitation centres

## Abstract

**Background:**

Substance abuse is increasing in South Africa with research indicating a lack of access to public drug rehabilitation centres in all provinces of the country. The insights of recovering substance abusers in terms of access to centres remain a gap in the north of Tshwane.

**Aim:**

The study explored the recovering substance abusers’ views or perspectives on the accessibility of long-term inpatient substance abuse rehabilitation centres.

**Setting:**

South African National Council on Alcoholism and Drug Dependence (SANCA) centers in Soshanguve and Hammanskraal.

**Methods:**

The qualitative study involved recovering substance abusers undergoing treatment at two outpatient substance rehabilitation centres in the north of Tshwane. The participants were interviewed using a semi-structured questionnaire which explored their views and perceptions and experiences on the accessibility to rehabilitation centres.

**Results:**

Saturation was reached at 13 male participants between 29 years and 35 years of age. The interviews were transcribed and translated, and five distinct themes were identified. The themes identified were: rehabilitation is mindset-related; long waiting times translated into continuous substance abuse; peer pressure; no jobs or skills to keep busy; and a lack of trust within the community. Four of the themes were attributed to enabling factors for their substance abuse.

**Conclusion:**

Long waiting times, few rehabilitation centres, stigma, the lack of jobs and skills were identified as barriers to access rehabilitation centres.

**Contribution:**

The study contributes to the body of literature exploring access problems in rehabilitation centres from the perspective of recovering drug addicts. It provides insights into risk factors that exacerbate the scourge of substance abuse in the north of Tshwane.

## Introduction

Substance abuse is defined by the Department of Social Development (2012) and the National Drug Master Plan (2013–2017) as the abuse of legal and illegal drugs, which includes alcohol, inhalants, over-the-counter drugs, prescribed drugs and indigenous plants. Mathibela and Skhosana (2019) state that substance abuse negatively impacts families, children and communities, and contributes to health challenges. Substance use is a major public health concern in South Africa (World Health Organization [WHO] 2014). The WHO estimated that around 7% of all deaths in South Africa are attributable to alcohol and drug abuse-related causes (WHO 2018).

The South African Medical Research Council (SAMRC) estimated that the direct costs (treatment, loss of productivity and costs related to criminal justice system) alone reached R37.9 billion, which was equivalent to 1.6% of the country’s gross domestic product (GDP) (South African Medical Research Council [SAMRC] 2020).

The demand for drugs across the African continent is expected to continue to rise, with some projections predicting 14 million drug users by 2050 (ENACT Africa 2019). Many individuals battling substance abuse do not have the willpower to stop on their own. Considering this increasing burden of substance abuse, there’s been a drastic increase in the establishment of private treatment services (Parry 2005). It is unfortunate that facilities aimed at rehabilitating individuals seem inadequate and are still not widely accessible to poorer communities (Ramlagan, Peltzer & Matseke 2010).

Factors shown to restrict treatment utilisation include affordability, limited awareness about where to seek help, geographic access barriers, stigma and social stigma within a person’s community (Tucker, Vuchinich & Rippens 2004).

In South Africa, a study conducted by Ramlagan et al. (2010), which explored the epidemiology of substance abuse treatment in South Africa, revealed that substance abuse treatment centres were mostly concentrated in 5 out of 9 provinces, namely Gauteng, Western Cape, Kwa-Zulu Natal, Eastern Cape and Mpumalanga, with an 83.1% demand from men alone. Access to these facilities is further complicated because demand does not match supply. The limited number of facilities does very little to meet the population’s needs. This is in line with findings by Mohasoa and Mokoena (2019), who suggest that some of the barriers to accessing substance abuse rehabilitation centres are long waiting lists for admissions and a limited number of rehabilitation centres. This occurrence is not unique to South Africa as limited substance abuse treatment and rehabilitation centres were also reported in Pakistan (Ghazal 2019), Kenya and Uganda (Janson et al. 2024) and the United States of America (Pullen & Oser 2014).

A study conducted in Tshwane, South Africa, found several barriers to treatment, including complicated admission procedures, a lack of awareness of services, stigma and the belief that treatment isn’t needed (Nyashanu & Visser 2022).

In addition to the above-stated factors that influence substance abuse and accessibility to treatment and treatment facilities are socio-economic factors, social and cultural factors, gender and race, policy and regulatory factors.

### Socio-economic factors

Substance abuse poses a major challenge among disadvantaged communities. Substance abuse, with particular emphasis on alcohol, is higher in informal settlements and townships compared to suburban areas (Mbandlwa & Dorasamy 2020). One of the reasons blamed for substance abuse is stress because of unemployment. Myers, Louw and Pasche (2010) and Janson et al. (2024) assert that cost and financial obstacles hinder access to treatment services.

Based on the above-mentioned studies, low socio-economic status could be a causative factor for substance abuse and an impediment to access treatment facilities.

### Social and cultural factors

Several socio-political factors have hampered access to treatment in South Africa. While seeking help is a good and necessary step, social stigma surrounding substance abuse may lead to delays in seeking treatment. Unless the environment where the substance abuser resides is supportive, individuals may not access help because of the lack of support and inadequate awareness levels. Stigma was identified as one of the most important barriers to treatment-seeking among young adults living with substance abuse. The negative perceptions of substance users in society lead those affected to believe they will face disapproval from family and friends, and therefore, they may be hesitant to seek treatment because of concerns about receiving proper care (Nyashanu & Visser 2022).

Given that South Africa is a multicultural country, the integration of religious, traditional and western approaches into treatment is imperative. It is important that there is collaboration between traditional health practitioners and primary health care services. Some people are of the opinion that beliefs in traditional medicine and healers (sangomas) are barriers to medical treatment, which is traditionally regarded as western treatment (Nyashanu & Visser 2022). Many people still rely on traditional healers rather than formal treatment services, which may be viewed with mistrust because of historical exclusion (Myers, Fakier & Louw 2009). Some families believe that religion, church and prayer are the ultimate solutions for any problem, including substance use. This perspective can sometimes hinder efforts to encourage individuals who use substances to enter formal treatment programmes (Nyashanu & Visser 2022).

The foregoing statements clearly show that culture, beliefs and religion are determinants that play an important role in access and utilisation of drug treatment centres. Anyone committed to fighting drug use needs to consider these points if their goal is to genuinely assist those who struggle with substance abuse.

### Gender and race

Males tend to use drugs more than females, to a ratio of 3:1, as they have more opportunity and funds compared to females. The lower usage in females is attributed, among other factors, to the fact that parents tend to be stricter to females as opposed to males (Ramlagan et al. 2010). A study conducted by Mokwena and Setshego (2021) also reported a higher substance abuse in men than women, and an increase of drug use in young people who start as early as 15 years. The issue with young people developing habits such as smoking cigarettes is that while it is legal for adults, it’s against the law for them at the age of 15. According to Ramlagan et al. (2010), drug abuse was more common among males, particularly Black or White individuals, who had lower levels of education. The study revealed that this demographic was disproportionately affected compared to females and people of other racial groups.

As far as access to substance abuse treatment is concerned, across countries, there is a reported under-representation of women and the racial profile that does not represent the demographics of the country in inpatient substance abuse treatment centres.

While women are underrepresented in substance use treatment, they face the same barriers as men – such as cost, transport, and limited awareness of options – but often with greater intensity, leading to consistently lower treatment rates for women (Janson et al. 2024). Myers and Parry (2005) highlighted how the race profile of clients at specialists’ treatment centres in Cape Town and Gauteng does not reflect the demographics of the general population. This finding should not be misinterpreted as indicating low levels of substance abuse; rather, it highlights the limited accessibility of treatment services to Black South Africans (Myers & Parry 2005).

### Policy and regulatory factors

Unlike other mental disorders that can be treated, addiction is a chronic relapsing mental disorder which requires structured treatment regimens and life-long management (Ghazal 2019). South Africa’s National Drug Master Plan (NDMP) and other policies aim to strengthen the fight against substance abuse and improve access to treatment. However, the impact of such policies seems insignificant as there is still easy access and poor regulation of substances (Mpanza & Govender 2017). The African Union (AU) has cited substance abuse as a primary challenge in achieving the United Nation’s (UN) Sustainable Development Goals (SDGs) and the AU Agenda 2063 (Janson et al. 2024).

## Link between sustainable development goals and access to long-term residential inpatient rehabilitation centres

This study aligns with SDG 3: Good Health and Well-Being, which seeks to secure healthy lives and promote well-being for all. Increasing access to rehabilitation centres contributes to the SDG 3 goal by improving well-being, lowering the health and social costs of substance dependence, facilitating recovery and reintegration into society (United Nations [UN] 2015). The emphasis on accessibility of long-term residential inpatient rehabilitation centres in the north of Tshwane also targets SDG 3.5, which is strengthening the prevention and treatment of substance abuse, including narcotic drug abuse and harmful use of alcohol. By evaluating accessibility, the study highlights the relevance of equitable health services and the need to reduce barriers to rehabilitation. Those battling with substance abuse require access to adequate prevention, treatment and rehabilitation resources. This study contributes to the overarching goal of minimising the impact of substance abuse on individuals and society, consequently bringing about healthier communities.

## Research methods and design

### Study design

The study adopted an exploratory, descriptive and contextual design, using qualitative methods. The study followed a qualitative phenomenological approach. The purpose of the phenomenological approach is to describe the essence of a phenomenon by exploring it from the perspective of those who experienced it in order to understand the meaning from their experience (Teherani et al. 2015).

### Setting

This study was conducted in the City of Tshwane in the following settings: The South African Council on Alcoholism and Drug Dependence (SANCA), in Soshanguve and Hammanskraal. The SANCA is an institute that offers outpatient prevention and treatment programmes for substance abuse to the community.

### Measurements

The measurement tool used was a semi-structured interview guide, which was audio recorded after consent was given for the recording. This semi-structured interview guide consisted of demographic questions and other questions with an opportunity to follow-up for clarifications purposes. This interview guide was self-administered by the principal researcher and one other researcher. The questions were open-ended and focused on the participants’ perceptions and experiences.

The following questions were asked during the interview:

- Which year did you start taking substances?
- Please list the substances you take.
- Is this the only facility you have received substance rehabilitation assistance from since you started seeking help?
- Describe to me how you got involved in taking/abusing substances.
- Tell me about how taking/abusing substances has affected you socially and physically within your community.
- Describe to me your experience of attending an out-patient rehabilitation centre?
- Tell me about your experiences and perceptions on the accessibility of long stay in-patient substance abuse rehabilitation centres.
- Have you ever been on any rehabilitation centre waiting list? If so, how long and what were you doing to cope while waiting?

### Sample size

The population recruited was recovering substance abusers who were undergoing treatment at the two selected substance rehabilitation centres – *SANCA in Soshanguve and Hammanskraal*. They were sober for at least 72 h and were over the age of 18. The researcher planned to interview 20 participants across the two study setting facilities, but data saturation was reached after the 13th interview.

### Data analysis

The researcher collected data through semi-structured interviews, and these interviews were recorded using an audio recorder. The interview was transcribed and translated, and themes were developed from the content of these interviews after agreement between the investigators.

### Trustworthiness

To ensure trustworthiness in this study, the researchers used Lincoln and Guba’s (2016) quality criteria framework, namely credibility, transferability, dependability and confirmability. The researcher ensured prolonged appointments with the participants in the field to extensively understand their context and triangulated data to establish credibility. Triangulation was ensured by collecting data using in-depth interviews and recording. Themes were developed after a discussion and engagement to sort out areas of differences. To ensure the findings’ transferability, the researcher extensively discussed the research methods and the context of the study. Dependability was maintained by holding regular correction and feedback meetings with the study’s supervisors. Confirmability was established through discussions between the researcher and their supervisors, who reviewed the findings until a consensus was reached.

### Ethical considerations

Ethical approval to conduct the study was obtained from the Research Ethics Committee of the Faculty of Health Sciences, University of Pretoria (Ref: 417/2023). No personal details of the patients were disclosed, and all information was strictly confidential and anonymous.

## Results

A total of 13 participants took part in the study. The ages of the participants ranged from 29 years to 35 years, and only males consented to participate. Six participants were from Hammanskraal SANCA, and seven were from the Soshanguve SANCA. Nine participants explained that they did not only seek help from the facility they were found at, but tried other facilities before they were accepted. Four participants were accepted by the facility where they were found and did not have to try other facilities before.

The participants’ first usage of substances ranged from 2003 to 2014. The majority of them used Nyaope with others using more than one substance. The following is a list of substances used with the number of the users in each category:

- Nine used Nyaope;
- Four used Crystal meth;
- Two used Weed/dagga (Cannabis);
- One each used Print (Fanya), Heroin, Cat (Methcathinone), Crack cocaine and Pash (white powder), respectively.

The following five themes emerged from the interview with the participants:

1. Rehabilitation is mindset related.
2. Rehabilitation centre waiting lists means continuous abuse and use of substances.
3. Peer pressure.
4. No jobs or skills to keep busy.
5. Lack of trust within the community.

### Rehabilitation is mindset related

Participants’ perceptions and experiences of the accessibility of inpatient long-stay rehabilitation centres were that the government has provided enough assistance in terms of making these facilities accessible and affordable. Furthermore, the participants kept referring to the importance of one’s mindset being ready to receive the help that may be provided to them. This means that regardless of being taken to a long-term inpatient rehabilitation centre, one needs to be mentally ready to embark on the journey to stop using or abusing substances. They alluded to the fact that one may come back from a long-stay inpatient rehabilitation centre and still relapse upon return to the community if they never made up their mind that they really wanted to stop using or abusing drugs:

‘Ya, they are easily accessible, what I can tell you is that the difficult thing is to open your heart, help is available to stop using nyaope, they should not lie to you, but if you have not made up your mind, like me maybe I went to rehab 7 times, 8 times but here I am smoking because I had not made up my mind, I was doing it to please people.’ (Participant A)

‘Well, yaa, nna, what I told myself is that there are some rehabilitation centres that are expensive, some don’t treat patients well, you get me, that’s why I don’t like some rehabilitation centres, rehabs that are affordable, because rehab is not like they take you there then you’re going to stop, nooo, it is about if you want to stop or not, there are a lot of others we’ve a lot of people being taken to rehab, after maybe a month or 2 months they come back and relapse, so it’s a waste of time, you see.’ (Participant B)

‘It’s not easy because you must work for it to get there, ya, you must prepare yourself tell yourself once things that you want then no one should force you. You need to take yourself, I took to rehab to myself, no one forced me, I saw that I was tired.’ (Participant C)

‘Its accessible but affordability depends on if it is a private one, but government is affordable, on the other hand its mindset related because one can come back from rehab the same due to not having made up their mind.’ (Participant D)

‘Rehab is up to you otherwise it’s a waste of time.’ (Participant E)

‘Rehabs are accessible but if you have not made up your mind you won’t leave.’ (Participant F)

‘… you need to work on yourself, it’s about mindset.’ (Participant G)

### Rehabilitation centre waiting lists are sometimes long which means continuous abuse or use of substances

The length of the waiting period and the effectiveness of the follow-up system were points of disagreement among participants. While some felt the wait was short, others found it to be long and the follow-up system to be weak. Some participants managed the waiting period by smoking. This is indicative of the systems that may be inconsistent in terms of getting the substance abuser the immediate help they need.

Participants had mixed experiences with waiting lists; some were contacted within 2–3 weeks, but others never received a response, possibly because of miscommunication. While waiting, many participants coped by continuing to use substances:

‘I have never been to a rehab but I was applying, but those people who were applying they just they go, they never came back, they just took our names, and addresses, and ID numbers, they said they will come and tell us when we can meet and they can arrange everything.’ (Participant F)

‘Ya, I was once on the waiting list, but there is no proper communications, they don’t follow-up at our homes, these phone call things ahhh.’ (Participant I)

‘I know some say they had to wait for 3 weeks.’ (Participant D)

‘I have never been on waiting list but they say it takes 2 to 5 weeks and people smoke while waiting.’ (Participant A)

‘I was on waiting list but they never contacted me since 2020.’ (Participant F)

‘Was on waiting list but the following up with phones doesn’t work, only if they’d fetch us physically …’ (Participant I)

### Peer pressure

In the interview, peer pressure consistently emerged as the primary factor contributing to substance abuse. The issue of trying to be part of a group and be accepted by friends lead them to abuse drugs, and for some, the lack of family support or love led them to seek such from peers:

‘By friends. Uhmm growing up, like bo stout [*being naughty*].’ (Participant A)

‘When I was young, my mother passed away, I was 9 years old, ya, somewhere there around 9 years, ya, eish so I lived most of my life in the street without parents’ love, like both parents, mom and dad, you get me, that’s what pushed me to smoke drugs.’ (Participant B)

‘Uhmm me, the thing that introduced me to substances, I could say was being forward, to belong, ya, the group that was smoking were my friends and it was friends I grew up with, I also ended up getting involved without knowing the side effects, in short, I wanted to belong.’ (Participant C)

‘Peer pressure got most of the substance user or abusers into the lifestyle.’ (Participant M)

‘Lived in the streets a lot without parents’ love.’ (Participant B)

‘… being forward … I wanted to belong.’ (Participant C)

### No jobs or skills to keep busy that lead to boredom

The participants’ experiences were that they lacked skills to keep busy which led to boredom and hence they resorted to substance abuse. The lack of jobs to keep them busy led them to abusing substances:

‘Sometimes, I need to be busy with something, have you noticed like I can leave and stay home, without having anything to do, staying, not having food, where will I go? To hustle. I am going to hustle in the street, when I get to the street, when I get to the street the mentality to get high comes back., you see, but if I get busy with a job, leaving the house in the morning and work until 5 or 6, then I can quit.’ (Participant B)

‘All about to be busy, that’s why you see me, I want to be busy, I don’t want to see myself not being busy, I don’t want to see myself in a place that seems slow as I get bored.’ (Participant D)

‘When sober there are withdrawals so I try to keep busy.’ (Participant D)

‘Government needs to provide us with jobs and skills after rehab in order to keep busy.’ (Participant J)

### Lack of trust within the community (stigma)

The participants expressed a shared sentiment that their addiction created a constant need for ‘the next fix’, yet they lacked jobs or a reliable income. The lack of money to buy substances prompted them to steal from their families and the community. Some of the substance abusers confessed that they were beaten up for stealing and experienced a lack of trust within the community. They felt nobody trusted them and there was this stigma attached to them as ‘thieves and useless people’:

‘Ah, it affected me a lot because community doesn’t trust me, and they once beat me 3 times for stealing and it affected me a lot, can’t exaggerate on how much but it affected me a lot.’ (Participant A)

‘Eish, it makes people to look down on you, they don’t take you seriously, ya, if you smoke they think maybe you’re a tsotsi, a criminal, they just don’t take you seriously.’ (Participant F)

‘… community doesn’t trust me because of stealing.’ (Participant B)

‘People don’t take you seriously because they think you’re a criminal.’ (Participant F)

‘Stealing becomes part of your identity for the next fix.’ (Participant J)

## Discussion

The study reached saturation at 13 male participants with no female volunteering to take part in the study. It would have been valuable to explore the challenges faced by female participants in the rehabilitation centres. Literature suggests that women’s exposure to various forms of violence – physical, emotional, and sexual – can push them into drug use (Boroumandfar, Kianpour & Afshari 2020). Other studies suggest that females have greater stigma attached to them if they abuse drugs and that they are discriminated against in rehabilitation centres, some even fear going for rehabilitation as they fear losing their children (Myers et al. 2009, 2010).

It was postulated that substance use prevalence for females may possibly be much higher but was underrated because of the fact that females were hardly seen in rehabilitation centres. This phenomenon suggests that females are concealed substance abusers.

The input from females in this study would have shed some light on their experiences and what specifically led them into substance abuse. This aspect is therefore missing in the study.

The study presented five themes which are discussed as follows.

In *theme 1*, the participants indicated that for a person abusing drugs to be rehabilitated, it is about a ‘mindset’. The participants explained that the substance abuser needs to be ready to change and must tell himself or herself that they are ready for rehabilitation. A study by Oguizu (2023) conducted in Nigeria agreed with the results of this study on this aspect. The study revealed that patients with substance abuse disorder weigh the pros and cons of drug use. Those who perceive more benefits than drawbacks are less likely to pursue drug-free living, often prioritising the perceived advantages and overlooking the negative consequences (Oguizu 2023). This is in line with the three stages of change readiness and treatment eagerness scale developed by Miller and Tonigan (1996). These are recognition, in which the patient acknowledges that they have a problem; ambivalence which indicates an openness to reflections, expected in the contemplation stage; and lastly, taking steps, indicating that the individual is already doing things to make a positive change in their substance use (Miller & Tonigan 1996).

The study’s participants felt that treatment facilities were accessible and affordable, explaining that this was because of free treatment at public health facilities. The participants also had different experiences in terms of waiting for admission to the drug facilities as some were admitted soon after they visited the drug facilities, while others were admitted after waiting for weeks and even worse, some never received any communication after their applications to the facilities. This concurs with findings from Sorsdahl et al. (2023), who mention that although public rehabilitation centres are affordable, accessibility is limited by factors such as long waiting times because of the unavailability of spaces. Public facilities also depend on government funding to provide services, and it is commonly known that they are not prioritised during budget-allocation. The limited allocation of funding to public health services results in overcrowded facilities, long waiting lists and limited access to comprehensive care (Sorsdahl et al. 2023).

The participants in the study experienced long waiting lists because the facilities were either full and in some cases were not even responding to applications. This is because of a limited number of rehabilitation centres, and all of these experiences align with findings from other studies (Mohasoa & Mokoena 2019; Nyashanu & Visser 2022 and Ramlagan et al. 2010). The circumstances as outlined then render treatment inaccessible to the addicts who desperately need the services.

The participants also mentioned that the longer they waited to access the centres, the more time they spent engaging in substances in order to cope, which is in line with *theme 2*. Masiko and Xinwa (2017) define substance abuse as the ‘persistent, overpowering, immoderate, and injurious use of substances, which comprises illicit drugs, alcohol, and prescription medication’, and this defines the state of affairs of these patients that desperately need help.

The participants in the study indicated that they began using substances because of peer pressure (*theme 3*), and this is not surprising as numerous studies have confirmed it (King 2021 and Reed & Rountree 1997). A commonly held view is that social pressure from friends to use drugs and alcohol is a major contributor to substance use (Reed & Rountree 1997).

The participants in this study complained that they did not have anything to occupy them and that boredom encouraged their drug use, which is in line with *theme 4*. This was in contrast to a study that was conducted among high school students investigating the association between leisure boredom and substance use (Wegner et al. 2006). However, a study conducted by Ziervogel et al. (1998) found that boredom has been found to be associated with substance use. Similarly, another systematic review indicated that periods of boredom increase impulsive decision making it plausible that individuals who are bored can easily decide to indulge in substances (Chao 2021). The study also concluded that experiences of boredom in chronic cocaine users are associated with worsened patterns of cocaine use.

The participants cited the lack of work and skills as a reason that lead them to abuse drugs. The issue of people working or not working has been found to be encouraging or discouraging for others to abuse drugs, and it also depends on the coping mechanism of the individual. Literature suggests a connection between work-related stress and drug abuse among some employees. However, others turn to drugs to cope with the stress of being unemployed (Henkel 2011). Literature also suggests that some unemployed people abuse less drugs because they don’t have the money to buy drugs and have no work-related stress (Henkel 2011).

Finally, the participants indicated that a lack of respect from their communities, seemingly because of an attached stigma, was a significant issue (*theme 5*). The participants felt that a primary reason for the stigma they faced is that they sometimes steal from their communities in order to get a fix. Understandably, communities want to prevent theft. However, when these communities don’t support substance users, it becomes a major hurdle. Without that crucial backing, it’s far less likely that substance abusers will be motivated to go to rehabilitation. This stigma has been found to be one of the important factors that prevents rehabilitation of the drug users. Because of the negative labelling in the community, people using substances anticipate rejection by their families and friends, and doubt whether they will receive appropriate healthcare from treatment centres (Nyashanu & Visser 2022).

### Recommendations

In alignment with the results of the study, it is recommended that provision of more treatment centres be prioritised to provide early intervention for people abusing drugs. Skill centres and extra-mural centres will keep people busy so that they can focus their energy levels on something productive.

Incorporating substance abuse awareness and stress management training into school curricula might be an effective strategy for prevention. Equipping learners with dangers of substance abuse and practical coping mechanisms may help reduce their vulnerability to substance abuse.

The Department of Health, the Department of Social Development, the South African Police Service and communities should collaborate and host more awareness drives in schools to halt this pandemic at its early stages. On higher level, funds should be prioritised and directed at preventing and providing interventions. Society and government must aim at increasing access to rehabilitation centres and therefore contribute to the SDG 3 goal by improving well-being, lowering the health and social costs of substance dependence, facilitating recovery and reintegration into society for substance abusers.

### Limitations

This study is not without limitations. Qualitative studies often have limitations such as small sample sizes and potential bias in answers. The sample size in this study was limited to 13 with only male participants. The experiences of female participants have been missed, which leaves a gap, as females often face violence and greater stigma than male participants. Despite the limitations, this study has provided some useful information.

## Conclusion

The participants initiated substance abuse as a result of peer pressure, lack of support from parents and community, and limited employment opportunities. Participants highlighted the difficulty of accessing rehabilitation centres because of their scarcity and long waiting times. They also mentioned facing social stigma from their communities, which created another barrier to seeking treatment. The long waiting lists contributed to continued substance abuse as individuals resorted to drugs as a coping mechanism.
